# A Long-Term Follow-Up of the Efficacy of Nature-Based Therapy for Adults Suffering from Stress-Related Illnesses on Levels of Healthcare Consumption and Sick-Leave Absence: A Randomized Controlled Trial

**DOI:** 10.3390/ijerph15010137

**Published:** 2018-01-15

**Authors:** Sus Sola Corazon, Patrik Karlsson Nyed, Ulrik Sidenius, Dorthe Varning Poulsen, Ulrika Karlsson Stigsdotter

**Affiliations:** Department of Geosciences and Natural Resource Management, Faculty of Science, University of Copenhagen, 1958 Frederiksberg C, Denmark; pakn@ign.ku.dk (P.K.N.); ulriksp@ign.ku.dk (U.S.); dvp@ign.ku.dk (D.V.P.); uks@ign.ku.dk (U.K.S.)

**Keywords:** stress-related illnesses, nature-based interventions, RCT, register data, pre-post study, health care utilization, therapy garden, CBT

## Abstract

Stress-related illnesses are a growing health problem in the Western world; which also has economic significance for society. As a consequence; there is a growing demand for effective treatments. The study investigates the long-term efficacy of the Nacadia^®^ nature-based therapy (NNBT) by comparing it to the efficacy of a validated cognitive behavioral therapy, called STreSS. The study is designed as a randomized controlled trial in which 84 participants are randomly allocated between the treatments. Long-term efficacy is investigated through data extracts from the national database of Statistics Denmark on the sick leave and the health-care consumption. The results show that both the NNBT and the STreSS lead to a significant decrease in number of contacts with a general practitioner in the period from twelve months prior to treatment to twelve months after treatment; and, a significant decrease in long-term sick leave from the month prior to treatment to twelve months after treatment. The positive long-term effects provide validation for the NNBT as an efficient treatment of stress-related illnesses.

## 1. Introduction

Stress-related illnesses are a growing health problem in the Western world [1,2], including Denmark [3]. It is estimated that around one-fourth of sick leave days in Denmark are due to stress [4] and 21.3% of the adult population report a high level of perceived stress [5]. Stress is not a disease in itself; but, in addition to affecting the person’s well-being and quality of life negatively, stress increases the risk of cardiovascular disease and mental illness, such as depression [6]. In addition to its effect on the individual’s health and quality of life, stress also has economic consequences for society since it affects productivity, leads to increased use of health services, increased sick-leave compensation, and early retirement [3]. One attempt to calculate the costs of stress in Denmark estimates that the annual cost is approximately DKK 14 billion [3]. This has led to an increased demand for interventions to prevent and treat stress-related symptoms. Different forms of cognitive behavioral therapy (CBT) are recommended by the Danish Health Authority [7]. Many other treatment forms also exist. One of the alternative treatments to CBT that has gained more public attention over the few past years is nature-based therapy (NBT). This is an umbrella term for a range of psychotherapeutic practices that integrate natural environments and nature-related activities into the therapy [8]. However, the field still relies to a large extent on qualitative research or quantitative research that lacks comparative groups [9]. To bolster the scientific standards of this therapy form, a randomized controlled trial (RCT) was conducted in the University of Copenhagen’s therapy garden, which is called Nacadia^®^. The aim of a previous study with respect to the RCT was to test the efficacy of the Nacadia^®^ nature-based therapy (NNBT), measured by mental health metrics regarding general psychological well-being and burnout. The NNBT was compared to a validated cognitive behavioral therapy known as STreSS (Specialized Treatment for Severe Bodily Distress Syndromes) [10]. The study found that the Nacadia^®^ NBT was equal in efficacy to the STreSS with regard to improving the subjects’ mental health status, measured from before to immediately after treatment [11]. Furthermore, the positive effect on mental health was found to be sustained a year after the treatment had ended [11]. Although the participants’ perceived mental health improved, the question remains whether the NNBT also had an effect on sick leave and healthcare consumption, thereby leading to fewer economic consequences for society. These questions are therefore the focus for the present study.

A study by Währborg and colleagues, which compared register data on health-care consumption one year prior to and one year after a Swedish NBT treatment, found that the NBT treatment led to a significant decrease in healthcare consumption, but no decrease in sick-leave compensation [12]. The study included comparisons with a matched population-based reference cohort receiving treatment as usual (not specified). No decrease in sick-leave or healthcare consumption was found for the reference cohort. Another Swedish study by Grahn and colleagues found that 44.2% of the patients had returned to work one year after a NBT treatment although only 14.7% on full-time basis [13]. This study did not include a control group and only measured self-reported return to work at two points in time: start of treatment and one year after treatment [13]. Due to the general lack of quantitative studies in the field of NBT—especially, studies with active control groups, the scientific evidence of its long-term effects may be considered very limited.

CBT is currently one of the most validated forms of psychotherapeutic treatment for stress-related illnesses [7]. According to systematic reviews, the scientific evidence for the efficacy of CBT with regard to decreased sick leave and return to work is moderate [14,15]. Nevertheless, CBT is generally found to be more effective when compared to other psychotherapeutic interventions or no treatment for improving mental health [14]. In the RCT design it was therefore decided to investigate the efficacy of the NNBT by comparing it to a validated CBT treatment (STreSS). The null hypothesis is that the NNBT will be as favourable as the STreSS, measured by long-term effects on sick leave and healthcare consumption.

Primary research questions:

- Is there change in participants’ sick-leave status from twelve months before a treatment to twelve months after the end of a treatment for the NNBT and the CBT, respectively?
- What trends can be observed in the participants’ number of contacts with a general practitioner twelve months before participation in a treatment to twelve months after the treatment has been completed for the NNBT and the CBT, respectively?

## 2. Methods 

### 2.1. Study Population 

In total, 200 potential participants were assessed for eligibility. Participants were referred to the project by municipalities, insurance companies, and health practitioners (private practicing doctors, psychologists, and psychiatrists). Some individuals also contacted the University of Copenhagen directly through brief notices in newspapers and on the university webpage that informed the public about the project. All of the potential participants receiving compensation benefits needed to provide authorization to participate from their municipal case officer. The assessment was conducted by one of four clinical psychologists and was supervised by a psychiatrist.

Inclusion criteria were that participants had to: (i) be between 20–60 years of age and fluent in Danish; (ii) incapacitated from work for at least three months prior to the start of treatment; and (iii) have a psychiatric diagnosis of adjustment disorder and/or reaction to severe stress as a primary diagnosis according to the International Classification of Disease F43 codes 0, 2–9 (acute stress reaction, adjustment disorders, other reactions to severe stress, and reaction to severe stress unspecified) [16]. Individuals with severe psychiatric morbidity, psychotic disorders, personality disorders, suicidal tendencies, and drug or alcohol problems were excluded.

The objective of the recruitment procedure was to identify and include participants who were so severely affected by stress that without treatment they would be at risk of developing a chronic stress condition and remain on sick leave. The aim of inclusion criteria (ii), being incapacitated from work for at least three months prior to the start of treatment, was aimed at excluding those who experienced acute stress but had recovered by themselves, as self-recovery usually occurs within a few weeks or months once the stressor is removed [16]. The recruitment procedure was as follows: existing relevant medical and psychological records (obtained with the subject’s consent) were sent to the project’s referring psychologists. The psychologists then assessed whether or not to call the individual in for an initial clarification interview. The clarification interviews were conducted by the project psychologists. They then conferred with the project psychiatrist in order to determine whether the potential participant met the inclusion and exclusion criteria to a reasonable degree. If necessary, the psychologists could recommend that the supervising psychiatrist assess an individual’s psychiatric suitability for the project (for more information on the recruitment process see online Supplementary Materials: Trial Protocol, Document S1). Adjustment disorders and reactions to severe stress have a range of different and mixed manifestations related to physiological, cognitive, and emotional functioning. Therefore, the included participants were not altogether homogenous concerning the manifestations of stress. However, overall, they shared the same symptoms of high arousal in the nervous system, depleted cognitive resources related to attention and memory, and emotional disturbance with different degrees of anxiety and depression.

Based on the inclusion and exclusion criteria, 84 participants were found eligible. They were evenly randomized to one of the two treatments (NBT = 43; CBT = 41). 

The study was designed as a randomized controlled trial (ClinicalTrials.gov ID NCT01849718) and followed the ethical principles of the World Medical Association’s Declaration of Helsinki (World Medical Association, 2013). It was approved by the Danish Data Protection Agency (J.No. 2013-54-0344) and by the National Committee on Health Research Ethics (P.No. H-1-2013-038). Participants received both oral and written information about the study and signed an informed consent statement before enrolment. 

### 2.2. Treatments

The NBT and CBT treatments took place simultaneously all year round from August 2013 to March 2015, except during holidays. Each treatment round lasted ten weeks. Recruitment of participants occurred from two months before the treatments started until the beginning of the last treatment period.

#### 2.2.1. The Nacadia^®^ Nature-Based Therapy

The NNBT took place at the University of Copenhagen’s therapy garden Nacadia^®^. It is located in an arboretum and designed as a 1.4-hectare wild forest garden [17]. The sessions lasted three hours and took place three times a week, during a ten-week treatment period. The NBT treatment may be categorized as a hybrid between individual and group-based therapy since most of the activities were individual, but the therapy took place in groups (mean group size = 6). Two licensed psychologists specializing in CBT and a gardener were involved in the sessions. The NNBT was developed by Corazon and colleagues [18]. It builds upon elements from mindfulness-based stress reduction and CBT, integrated with theories from environmental psychology [19].

The NNBT consists of five interrelated components: (i) individual therapeutic conversations based on CBT; (ii) individual and group mindfulness exercises, such as mindful walking in the garden; (iii) individual and social gardening activities, depending on the season, which integrates training in mindful awareness; (iv) individual relaxation and reflection time in the garden; and (v) homework to practice the techniques introduced. 

#### 2.2.2. The Cognitive Behavioral Therapy STreSS

The CBT took place indoors at the clinics of private licensed psychologists specializing in CBT. The treatment lasted ten weeks with 20 individual sessions, each of which lasted one hour. The therapy was based on a form of CBT, called Specialized Treatment for Severe Bodily Distress Syndromes (STreSS) [20]. The STreSS treatment consists of nine modules of manualized CBT, and its efficacy has been tested in a randomized controlled trial [10]. 

#### 2.2.3. Support to Return to Work

Both treatments entailed conversations between the patient and the therapist that focused on identifying opportunities for and challenges in returning to work or finding a job after the end of treatment. This process started in week six of the treatments. The therapists collaborated with employers to enable participants who were employed but on sick leave to return to work. For those participants without employment, the therapists collaborated with case officers and job consultants on work placement or other work-related initiatives after the end of treatment. 

### 2.3. Outcome Measures and Data Retrieval

The outcome measures were (i) sick leave and (ii) number of contacts with a general practitioner (GP). The data was extracted from the national database of Statistics Denmark. Due to the sensitive nature of the data, the participants were anonymized in the extracted data.

#### 2.3.1. Sick Leave

In Denmark, social insurance is an integral part of the public social security system. If a citizen falls ill while employed or unemployed, he/she is entitled to sick-leave compensation. This also applies in the case of adjustment disorders and/or acute and severe stress. Students, however, are not entitled to sick-leave compensation, but may apply to have their education allowance extended due to long-term sick leave. In Denmark, sick-leave compensation is generally time-limited to 22 weeks, but can be prolonged to a maximum of two and a half years based on an assessment of the individual case. After an initial 22 weeks of receiving sick-leave compensation, the citizen’s situation is evaluated by the municipality and the compensation is either terminated or extended based on the extension rule in the social security act.

Data on sick leave were retrieved from the register: AMRUN; variable: SOC_STATUS_KODE, which provides information on a person’s socioeconomic status. Extractions were based on the following codes: No. 317 (Sick leave from employment) and No. 612 (Sick leave from unemployment). (Student sick leave is not registered).

#### 2.3.2. Healthcare Consumption

All of the services provided by general practitioners are covered by the Danish social security system. Data on number of contacts with a GP were retrieved from the register: SSSY, which provides information on health care benefits received. Data were extracted from the variable SPEC2, code No. 80, which documents services provided by a GP (face-to-face consultation with the GP, and telephone or online consultation). 

#### 2.3.3. Background Characteristics

The participants completed a background questionnaire at treatment start. The background questionnaire contained questions regarding age, gender, demography, educational level, current employment status, and changes in employment status during the last 12 months. 

### 2.4. Statistical Methods

The Shapiro-Wilk test, skewness and kurtosis values, together with a visual inspection of the histograms and the box plots of the sample distributions, were used to detect any deviations from a normal distribution. The non-parametric Levine’s test [21] was used to reveal any heterogeneity of variance between the distributions before and after the treatment. The Wilcoxon signed-rank test was performed for the corresponding comparison of the number of months of sick leave within the twelve-month period before treatment, and the twelve months after treatment for the NBT and the CBT treatments, respectively. The McNemar’s test [22] was used to study any changes in sick leave among the participants in the period between the month before the treatment started and twelve months after the treatment had ended for the NBT and the CBT treatments, respectively. The Wilcoxon signed-rank test [23] was used to compare the number of contacts with a general practitioner during a period of 12 months before and 12 months after the treatment.

## 3. Results

**Figure 1:**
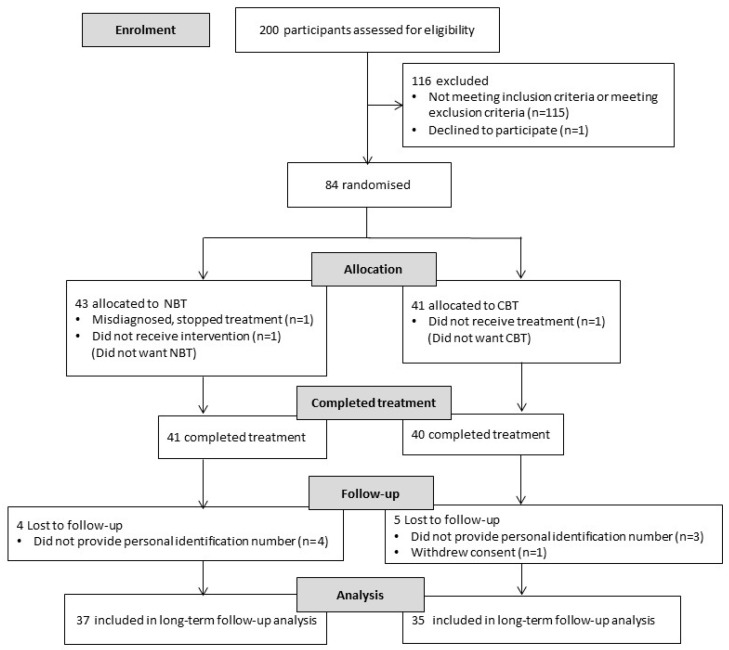
Participant flow diagram.

Of the 83 participants allocated between the two treatments, 41 participants completed the NBT treatment and 40 participants completed the CBT treatment (see Figure 1). Four participants in the NBT were lost to follow-up because the correct personal identification number was not provided. Five participants were lost to follow-up in the CBT; four participants did not provide the correct personal identification number; and, one participant withdrew consent after the treatment.

The results from the background questionnaire showed that the vast majority of participants were women (NBT: 82%, CBT: 82%) with a mean age of 47.9 in NBT and 44.9 in CBT [11]. The majority had a university bachelor degree or higher education (NBT: 76%, CBT: 68%). The categorization of participants with respect to socioeconomic status showed an even distribution between the two treatments; the majority of participants in both NBT and CBT were employed at the start of treatment (NBT = 20; CBT = 21), and a great number held a top-level position (NBT = 13; CBT = 12) (Table 1). A substantial proportion of participants were also registered as unemployed at treatment start (NBT = 18; CBT = 18). Of those, 17 participants from the NBT and 16 participants from the CBT were fired from work during the twelve months preceding the start of treatment. Moreover, one participant in the NBT and two participants in the CBT quit their job during that period.

### 3.1. Sick Leave 

**Figure 2:**
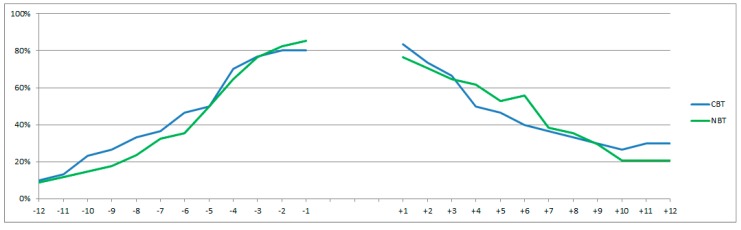
The proportion of participants on sick leave from twelve months prior to twelve months after a treatment. (NBT: *n* = 34; CBT: *n* = 30). Note 1. The gap in the graph represents the treatment period; Note 2. Data from the register: AMRUN; variable; SOC_STATUS_KODE was not available for 2016; therefore, data on three participants in NBT and five participants in CBT are missing.

All of the participants were rated as incapacitated for work for at least three months prior to treatment start, as stated in the inclusion criteria, but not all participants were registered as being on sick leave in Statistics Denmark prior to treatment start (Figure 2). They were, instead, registered as receiving other rehabilitation benefits or as students. 

The sample distributions, representing the number of months of sick leave, expressed significant deviations from the normal distribution (*p* < 0.05). Efforts to establish normal distributions using various types of statistical transformations were unsuccessful.

No significant change was found in the total number of months of sick leave for the participants in the period from twelve months before the treatment compared to the twelve months after the treatment for either the NBT or the CBT treatments (*p* > 0.05). Figure 2 illustrates the continous increase in the number of participants on sick-leave in the twelve months up to the start of treatment and, similarly, the similar decrease in the number of participants on sick leave from the end of the treatment to twelve months after.

A significant decrease was found between the participants on sick leave one month prior to treatment and in month twelve after treatment (NBT: *p* < 0.001; CBT: *p* < 0.01), (Table 2).

23 participants (77%) who were on sick leave prior to treatment and received the NBT treatment were not on sick leave twelve months after completing the treatment (Table 2). 15 participants (60%) who were on sick leave prior to treatment start and received the CBT treatment were not on sick leave twelve months after completing the treatment (Table 2). In total, 27 participants from the NBT group and 20 participants from the CBT group were not on sick leave after completing the treatment.

### 3.2. Healthcare Consumption

For the NBT and the CBT, there was a significant reduction in number of contacts with a GP twelve months after treatment as compared to the twelve months before treatment (NBT *p* ˂ 0.01; CBT *p* ˂ 0.05) (Table 3). The sizes of the effect were r = −0.396 (NBT) and r = −0.249 (CBT).

In terms of the number of contacts, the median number of contacts with a GP is still much higher for the participants one year after the treatment for both the NBT and the CBT groups when compared to the adult population of Denmark as a whole (mean number of contacts = 5.95) [24]. (Because the sample did not show a normal distribution, it was decided to use the median value instead of the mean value, which is used in the national sample. Nevertheless, the two statistical measures are still comparable in terms of understanding the central tendency of the statistical scores).

## 4. Discussion

Both the NBT and the CBT treatment showed similar significant long-term effects in the form of less healthcare consumption measured as the number of contacts with a GP one year prior to treatment as compared to one year after treatment. The decline in number of contacts with a GP after the NBT treatment are in line with the Swedish study by Währborg and colleagues mentioned in the introduction [12]. In the Swedish study, the participants’ number of contacts with a GP fell to national level one year after treatment. In this study, the number of contacts with a GP one year after treatment was still approximately two to three times higher for the NBT and CBT participants compared to the adult population as a whole. Thus, the participants in the NBT and the CBT still caused greater healthcare costs in regard to this variable when compared to the general population. It was decided to focus only on the variable “number of contacts with a GP” concerning healthcare consumption, because this was stated by the participants as their most common personal re-habilitation initiative prior to treatment [11]. No participants were hospitalized prior to treatment due to stress-related illness. Therefore, this variable was not investigated. 

The participants showed significant sick-leave reduction after receiving either the NBT or the CBT treatment. These results differ from the study by Währborg and colleagues, which also made use of register data and found no change in sick leave consumption in an identical period [12]. The other Swedish NBT study by Grahn and colleagues found an increase in return to work [13]. This study relied on self-reported measures. The disadvantage of using register data in a study like this one is that it cannot embrace nuances in a person’s employment status. For example, participants may have returned to work part-time, but are registered in terms of their primary status, which may be “on sick-leave”. A study such as the one by Grahn and colleagues may capture those nuances in self-reports. On the other hand, the risk of bias is higher when one relies on self-reporting rather than register data [25]. In the present study, we chose the variable “sick leave” instead of “return to work” in order to capture the status of those being unemployed. However, there are many confounding factors that are related to employment and sick leave registration, which is also mentioned as a limitation in similar studies [12,26]. Many gray zones exist between changing from sick leave compensation to other compensation services, while still being unavailable on the labor marked. This is also a limitation to the present study since it is not possible to deduce whether participants who were not registered as on sick leave were actually available on the labor market. In Denmark, sick-leave compensation generally terminates after a certain period, but may be prolonged based on the assessment of the individual case [27]. After termination of sick-leave, the person, if unemployed, will usually be transferred to job training or, in rarer cases, early retirement. These changes in status are hard to detect in registers. Therefore, the results only represent a part of the full picture. This is also why it is quite difficult to estimate the social costs of stress related illness. A recommendation for future studies, therefore, is to use both self-reports and register data and to follow the participants for several years. Thus, it would be possible more fully to capture the nuances between sick leave and return to work as well as long-term societal costs of stress-related illness, such as early retirement.

Although the treatments in the two Swedish studies were also NBT-based, the various NBT treatments currently on the market differ substantially in therapeutic method and content [9]. Consequently, the results from one study on the Nacadia NBT cannot be generalized to the entire field of NBT for stress-related illnesses. 

Review studies conclude that the “return to work” rate from any type of rehabilitation related to mental and physical health issues is low if they are not implemented at an early stage [28,29]. A Danish three-year follow-up study on a stress treatment program, which included 223 individuals on sick leave, reached the same conclusion; the length of sick leave prior to treatment is a predictor of the “return to work” rate [26]. In the present study, the decline in sick leave after the treatments may be due, besides the treatments’ efficacy or self-recovery, to the implementation of the treatments at an early stage. The results from the present and prior studies, therefore, highlight the need to identify work-related stress at an early stage and to launch effective prevention and treatment initiatives before the condition becomes chronic.

The mental health effects of the NNBT and the CBT treatment (published prior to this study [11]) showed significant improvements in participants’ perceived psychological well-being and a decrease in burnout after the end of both treatments, which was sustained a year after. In estimating the efficacy of stress treatments, it is common to look only at the immediate mental health benefits, leaving out the long-term effects relating to, for example, the reduction of sick leave [14]. Here, investigating both variables provides a more valid picture of the efficacy of the treatment.

## 5. Conclusions

The positive long-term effects in terms of a reduction in the number of contacts with a general practitioner and sick leave at twelve months after treatment provide validation to both the Nacadia^®^ NBT and the cognitive-behavioral STreSS treatment as efficient for stress-related illnesses. Having a randomized controlled trial with long-term follow-up in the field of NBT provides a good opportunity to provide scientific recognition for the field, which until recently has relied mostly on qualitative research. 

## Figures and Tables

**Table 1:** Socioeconomic classification of participants.

Socioeconomic Classification	NBT (*n* = 37)	CBT (*n* = 35)
Employee—ground level	2	5
Employee—intermediate level	3	3
Employee—top level	13	12
Chief executive	1	0
Self-employed	1	1
Unemployed	18	18
Student	2	3
Other compensation services/job training	3	0

Note 1. Classification based on the register: AMRUN; variable; SOC_STATUS_KODE; Note 2. Some participants were registered under several codes in the AMRUN register at treatment start (NBT = 6 participants, CBT = 7 participants).

**Table 2:** Participants’ sick-leave status from the month prior to treatment compared to twelve months after the treatment (NBT: *n* = 34; CBT: *n* = 30).

Treatments	Point in Time	Sick-Leave Status	Month 12 after the Treatment	
			Not on Sick Leave	On Sick Leave
NBT (*n* = 34)	1 month before the treatment *	Not on sick leave	4	0
		On sick leave	23	7
CBT (*n* = 30)	1 month before the treatment *	Not on sick leave	5	1
		On sick leave	15	9

* As the AMRUN register data is only available on a monthly basis, the starting point for the analysis was decided to be the month prior to the date of the treatment start; Note 2. Data from the register: AMRUN; variable; SOC_STATUS_KODE was not available for 2016; therefore, data on three participants in NBT and five participants in CBT are missing.

**Table 3:** Number of contacts with a general practitioner from twelve months before treatment to twelve months after treatment (NBT: *n* = 37; CBT: *n* = 35).

Treatment	Point in Time	Median	Max.	Min.	Sign.
NBT (*n* = 37)	Before	18	42	1	*p* = 0.001 **
	After	13	67	0	
CBT (*n* = 35)	Before	21	103	6	*p* = 0.04 *
	After	14	35	5	

* *p* < 0.05; ** *p* < 0.01.

## Supplementary Materials

The following are available online at www.mdpi.com/1660-4601/15/1/137/s1, Document S1: Trial Protocol.
