# Supplementary material for: A Long-Term Follow-Up of the Efficacy of Nature-Based Therapy for Adults Suffering from Stress-Related Illnesses on Levels of Healthcare Consumption and Sick-Leave Absence: A Randomized Controlled Trial
Source: Int J Environ Res Public Health. 2018 Jan 15;15(1):137. doi: 10.3390/ijerph15010137 (PMC5800236; doi:10.3390/ijerph15010137)
Supplement: Supplementary file 1 [file ijerph-15-00137-s001.pdf]

Supplementary Materials: A Long-Term Follow-Up of the Efficacy of Nature-Based Therapy for Adults Suffering from Stress-Related Illnesses on Levels of Healthcare Consumption and Sick-Leave Absence: A Randomized Controlled Trial

Sus Sola Corazon, Patrik Karlsson Nyed, Ulrik Sidenius, Dorthe Varning Poulsen and Ulrika Karlsson Stigsdotter

Project Description: Nacadia Effect Study—NEST—Updated Project Description, April 2013 (A Previous Version Has the Tryg Foundation Case No. 7206-08)

Contents

1. Summary

2. The Purpose of the Project

2.1. Overall Hypotheses

2.2. Hypotheses Specific to the Nacadia Treatment

2.3. Success Parameters for the Project

3. Literature Review

3.1. Natural Environments and Garden Therapy

3.2. Alnarp Rehabilitation Garden at the Swedish University of Agricultural Sciences

4. The NEST Setup

4.1. Similar Factors in the Two Types of Treatment

4.2. Divergent Factors in the Two Types of Treatment

5. Inclusion and Exclusion Criteria

Selection Procedure

6. Timetable for the Treatment

7. Data Acquisition and Research Methodology

7.1. Primary Outcomes

7.2. Forms

7.3. Databases

7.4. Statistical Considerations

7.5. Explorative Data Acquisition in Relation to Nacadia Treatment

7.6. Ethical Considerations

7.7. Expected Benefits for Test Subjects

7.8. Risks

Process for Obtaining the Subjects' Consent

7.9. Insurance

8. Timetable for the Research

8.1. Timetable for the Comparative Research Component

8.2. Timetable for the Longitudinal Research Component

8.3. Timetable for the Explorative Research Component

8.4. Communication of the Outcomes

8.5. Relevance and Target Group

9. Project Organisation

References

1. Summary

In the Western world, stress is considered as one of the most important factors in relation to modern lifestyle ailments. This research project deals with the treatment of stress in the Nacadia therapy garden (hereinafter Nacadia therapy), see Figure S1. The purpose of the Nacadia garden is to serve as a full-scale laboratory for research into the effect of garden therapy and the design of therapy gardens, as well as being a "demonstratorium" and a teaching facility within the field. Nacadia is located in the Arboretum in Hørsholm. The garden has an evidence-based design that I specially devised for the treatment of the target group concerned. Nacadia therapy is based on the assumption that the patient's health and well-being will be improved by spending time in a natural environment and participating in meaningful gardening activities. The therapy consists of individual and group activities in the garden (both physical and mental), e.g., gardening, walking, rest, and talk therapy.

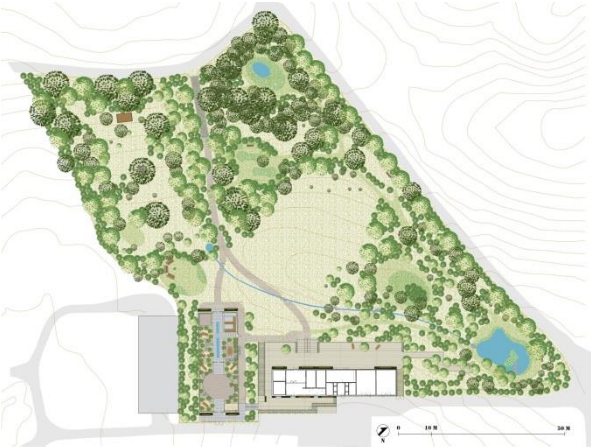

Figure S1. The Nacadia Therapy Garden.

The purpose of the NEST project is to conduct research into the effect of Nacadia therapy. It consists of a randomised controlled study, including longitudinal studies comparing the effects of Nacadia therapy and cognitive behavioural therapy (CBT) for the groups.

Test subjects will be recruited from private GPs, insurance companies and job centres run by neighbouring local authorities. The main criteria for inclusion are that the subjects:

- are suffering from long-term stress and are off work due to long-term stress;
- do not suffer from other significant and untreated underlying physical disorders;
- do not have other significant and untreated underlying mental disorders, e.g., personality disorders, bipolar disorders, psychosis or a high degree of social phobia; and,

- are not suicidal or an abuser of drugs or alcohol.

Subjects will be informed that it may be a demanding process (with potential risks), which will often require subjects to challenge themselves mentally/psychologically, and that they may at times falter or regress.

The outcome indicators include frequency of return to work, frequency of use of the healthcare system, psychological indicators that are related to stress, health, quality of life, and changes in medicine use. Explorative studies will also be conducted, in the form of observations and interviews, in order to obtain a better understanding of the gardens' and garden activities' importance to the therapeutic process.

The Tryg Foundation is providing DKK 3 million in financial support for this three-year project.The therapy garden cost DKK 6,350,000.

The project results will be communicated to the international academic community via articles in scientific journals and presentations at international conferences. They will also be conveyed to the Danish public via popular-science articles and reports.

Ethical approval of the project has been sought from the Scientific Ethical Committee, Capital Region of Denmark.

The Department of Geosciences and Natural Resource Management (IGN) is authorised to conduct register-based research, and issue permits and notifications concerning the collection of sensitive personal data will be submitted to Statistics Denmark. The project has been registered with and approved by the Danish Data Protection Agency, which in accordance with the Data Protection Act, permits the acquisition and processing of sensitive personal data for purely scientific or statistical purposes. The information provided to the test subjects and the processing of personal data will be in accordance with the Data Protection Agency's terms and conditions for private research projects in relation to the duty of disclosure, subject insight and options for citing objections, as well as the retention and deletion of data. Via the consent form, the subjects give their consent to the collection and processing of sensitive personal data.

2. The Purpose of the Project

The purpose of the project is to carry out research into the effect of Nacadia therapy compared with CBT, in order to gain an insight into the therapeutic potential of this type of treatment for people with stress-related disorders. CBT is the type of treatment with the greatest amount of evidence for its effect on stress-related conditions, such as anxiety and depression. Along with mindfulness, CBT has been shown to be the most effective form of therapy for treating stress. CBT is therefore the most widespread psychotherapeutic type of treatment, both in psychiatry and in general practice. The control group for this project will be treated on the basis of Andreas Schrøder's CBT manual, which has been especially designed for this target group and has a proven track record of good results. The Nacadia therapy will therefore be compared with the best existing evidence-based form of treatment. Several important elements of the project design are identical to those that are used at the Swedish Agricultural University (SLU) in Alnarp. In the long term, this will make it possible to compare the results in subsequent research projects.

2.1. General Hypotheses

1. Research has shown a correlation between human health and attending and engaging in activities in specific types of natural environment, especially in relation to stress [1,2]. This forms the basis for the project's hypothesis that garden therapy in a specially designed natural environment will lead to improved health and well-being for people on sick leave with stress-related disorders.
2. One underlying hypothesis is that the design of the therapy garden not only supports its use and accessibility, but also directly boosts its health-promoting qualities by facilitating restorative outdoor pursuits. This hypothesis is based on knowledge about how design

influences human well-being [3], which is related to the field of "health design" and the landscape architecture movement known as "healing gardens" [4,5].

2.2. Hypotheses Specific to Nacadia Therapy

1. The season of the year makes no significant difference to the effects of Nacadia therapy.
2. The patient's age, gender or socio-economic background make no significant difference to the effects of Nacadia therapy.
3. After the treatment, the patient's quality of life will be significantly enhanced.

2.3. Success Parameters for the Project

- Measured on the basis of psychological parameters, there will be no significant difference between the effect of Nacadia therapy and the effect of individual CBT.
- There will be no significant difference in frequency of "return to work" and "use of the healthcare system" between the Nacadia therapy group and the CBT group, measured on completion of treatment and three, six, and 12 months later.

3. Literature Review

A growing number of studies are evaluating the effectiveness of interventions in relation to stress treatment. Studies focusing on work-related stress demonstrate the effectiveness of using CBT and multi-faceted therapy, but find only a moderate effect from the exclusive use of relaxation exercises [6-11]. Symptoms and job satisfaction are the most frequently used outcome measures in the studies. Other studies have demonstrated an effect in relation to physiological parameters [12,13]. In relation to the use of sick leave as an outcome measure, Dutch and Danish interventions have shown a moderate effect [12,14,15]. An intervention at *Stressmottagningen* (The Stress Reception) in Stockholm only showed the effect in comparison with those who did not receive other treatment [16].

3.1. Natural Environments and Garden Therapy

There is growing evidence that spending time in and looking out on specific types of natural environments can have a stress-reducing and health-promoting effect, among other things by influencing our nervous system, cognitive resources, and mental well-being [2]. This research forms the basis for outdoor therapy and for therapy gardens, which are deliberately designed to support rehabilitation processes for specific patient/client groups. Garden therapy takes place in a designed natural environment and falls under the broader category of nature-assisted therapy. This consists of therapeutic and rehabilitative interventions that involve plants, natural materials and/or outdoor environments [1].

Research into the effect of nature-assisted therapy is still in its infancy, and there is a lack of quantitative, comparative, and longitudinal studies [3]. The studies have also been carried out on different patient/client groups and diverging approaches to treatment, which makes it difficult to compare them, draw conclusions about their efficacy and understand the mechanisms that trigger the effects. A review study from 2011 took stock of research into nature-assisted therapy. The results of this study show moderate evidence for the effect of nature-assisted therapy across patient groups and approaches to treatment [1].

It should be noted that there has been an increase in the volume of research into nature-assisted therapy in recent years, especially in Scandinavia. For example, two Swedish and Norwegian PhD theses have been written on the mechanisms in and the effect of nature-assisted therapy. These studies suggest that there is a positive effect in relation to the treatment of people with stress and depression [1,17,18].

3.2. Alnarp Rehabilitation Garden at the Swedish University of Agricultural Sciences

SLU in Alnarp, Sweden (see Figure S2) is one of the world's most renowned providers of garden therapy for people that are affected by stress (for a description of the treatment, see [19]. A study of 120 garden-therapy patients shows that 89% reported improved quality of life (measured by four standardised instruments). On average, the improvement was 20–40%. These results have not yet been published, but they were reported by the head of research, Professor Patrik Kittel Grahn.

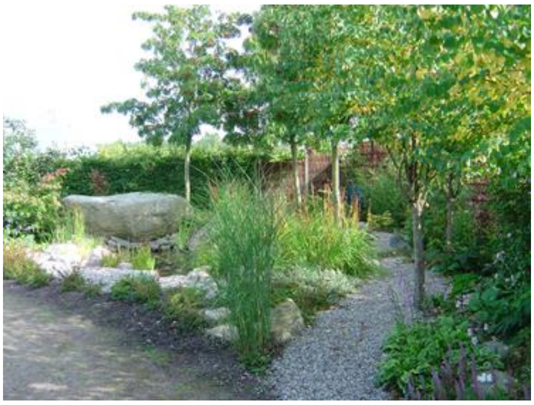

Figure S2. The therapy garden in Alnarp.

4. The NEST Setup

To enhance the quality of the project's evidence-based research, the intervention takes the form of a randomised controlled study that compares two types of treatment by taking repeated measurements of the same variable over a prolonged period. Experience of previous studies shows that it is not possible to include a control group of patients who receive no treatment, e.g., people on waiting lists, as people suffering from stress will generally seek their own treatment if they are not offered a place on the project. In addition, it is not considered ethically responsible to include a "non-treatment group" [12].

The types of treatment used in this study are Nacadia therapy (garden therapy) and CBT (the control group) (see Table S1). Due to the fact that Nacadia therapy is takes many hours of effort, it will be compared with the longest conventional treatment available via the national health service for stress-related conditions such as anxiety and depression.

Table S1. The two types of treatment used in the project.

Treatment: (*n*) 80 Patients in Total		
**Types of Treatment**	**Nacadia Therapy [1]—(*n*) 40 Subjects**	**CBT [2]—(*n*) 40 Subjects**
Environment	Nacadia therapy garden (outdoors or in greenhouse)	Clinic (indoors)
Therapists and other staff	2 psychologists (garden therapists) and a gardener	2 psychologists
Length of treatment	10 weeks	10 weeks
Content of treatment	96 h of Nacadia therapy, which includes:<br>79 h of garden therapy<br>10 × ½-h individual conversations<br>4 × 3-h exit interviews	20 h of individual CBT, which includes:<br>16 h of treatment<br>4 × 1-h exit interviews
Treatment set-up	Groups of 8 people	Individual

[1] The Nacadia therapy is described in detail in Appendix A; [2] Cognitive Behavioural Therapy is described in detail in Appendix B.

4.1. Similar Factors in the Two Types of Treatment

- *Therapists:* Authorised psychologists provide both forms of treatment. The psychologists are trained in CBT.
- *Talk therapy:* For both types of treatment, the psychotherapy is primarily based on CBT.
- *Length*: Both types of treatment last for 10 weeks

4.2. Diverging Factors between the Two Forms of Treatment

- *The environment:* In Nacadia therapy, the treatment is provided in a specially designed natural environment. In CBT, it is provided exclusively in the psychologists' treatment rooms.
- *Number of hours*: In Nacadia therapy, the individual sessions last three hours, with two sessions a week during the first and last weeks, and three sessions per week in weeks 2–9. This makes a total of 96 h, including 4 × 3-h exit interviews/follow-up and 10 × ½-h individual sessions. In CBT, the individual sessions last an hour, with 1–2 sessions per week. This makes a total of 20 h, including 4 h of exit interviews.
- *Content of the treatment*: Nacadia therapy uses garden therapy, i.e., things to see and do outdoors, integrated with mindfulness training. The individual interviews in the Nacadia therapy will be mindfulness-based CBT; for the control group, they will be CBT.
- *Number of hours of talk therapy:* The Nacadia therapy includes 10 × ½-h sessions of individual psychotherapy. For CBT, the individual psychotherapy amounts to 20 h.

5. Inclusion and Exclusion Criteria

The project (IGN) has concluded agreements with a number of the surrounding local authorities to refer test subjects aged 20–60 to the project via the rehabilitation institutions Incita and Café Væksthuset. These are external stakeholders that are linked to local authority job centres. People in the area covered by the local authority can take part in the project if they are referred by their own doctor/psychiatrist or if they are clients of Topdanmark. They will receive detailed information about the project, the target group and the procedure for initial clarification.

Stress is the human biological response to pressure and demands. Stress is not a problem in itself. However, human responses to it are crucial. Stress reactions affect both the body and the mind, e.g., the nervous system, muscles, blood pressure, blood circulation, hormones, and concentration. If you are exposed to long-term stress with no option for rest to allow the body time to recover, this may result in both physical and emotional problems [20].

The project targets people suffering from severe stress (including the ICD categories F 43.0-9 (minus 1 = PTSD), and F45.3), which corresponds to being on sick leave for 6–24 months. Other treatment options do not usually last as long and have regular exit points, and are therefore more appropriate for people who are less severely stressed than those targeted by this project. The treatment takes 10 weeks, and any subsequent gradual reintegration into a job or work placement adds extra weeks or months. The partners, the project's psychologists, and those responsible for the medical condition of the subjects must therefore be aware that the period of sick leave must not be scheduled to end during the treatment period. It is difficult to give an unambiguous answer as to how long a person suffering from stress needs to be on sick leave, since it depends on several factors that can be difficult to map. In addition, it is quite common for stressed employees to take sick leave several times. The project's partners, referring psychologists and those that are responsible for the medical condition of the subjects are therefore aware of this problem. As a result, they try to identify and refer people who are so severely affected by stress that without treatment they would probably remain on sick leave for a minimum of a further three months.

For the purposes of the project, sick leave of 6–24 months is considered long-term—and therefore, an indicator that the individual is suffering from severe stress. However, even after relatively short-term sick leave (min. three months), the psychologists and the doctor/psychiatrist responsible for the treatment have the opportunity to make an assessment. The clarification interview with the patient, combined with a review of their medical and psychological records (obtained with the patient's consent) provide relevant insight into the subjects' medical and sick-leave history as well as their various stress symptoms. This can form the basis for assessing that someone who has been on sick leave for less than six months (but at least three months) has significant symptoms of severe stress and is therefore suitable for participation in the project.

The partners may refer people to project if:

1. their own doctor/local authority GP/pension provider's doctor/psychiatrist is of the considered opinion:
    - that they are suffering from long-term stress (sick leave for (3) 6–24 months);
    - do not have other significant and untreated underlying mental disorders, e.g., personality disorders, bipolar disorders, psychosis or a high degree of social phobia; and,
    - are not suicidal or an abuser of drugs or alcohol.
2. the partners, the local council case worker, psychologist or doctor/psychiatrist thinks that they:
    - have a prospect of returning to work but have been on sick leave for (3) 6–24 months;
    - are able to get themselves to garden therapy three mornings per week and actively participate;
    - are prepared to accept that it may be a demanding process that often requires subjects to challenge themselves mentally/psychologically, and that they may at times falter or regress;
    - are not about to undergo any major socio-psychological event, such as divorce;
    - are prepared to spend most of the time outdoors throughout the year;
    - are able to be part of a group (not too high a degree of social phobia);
    - are prepared to practice mindfulness through meditation and yoga, and spend up to an hour every day doing physical exercise at home;
    - are aware that they may be allocated to either garden therapy or individual cognitive therapy; and,
    - are prepared to complete a set of questionnaires four or five times in the course of a year, and be interviewed by researchers approx. five times.

5.1. Selection Procedure

The partners send (see Exclusion 1) the existing relevant medical and psychological records (obtained with the subject's consent) to the project's referring psychologists. The psychologists then assess whether or not to call the individual in for an initial clarification interview.

The clarification interviews (see Exclusion 2) are conducted by the project psychologists in Hørsholm and Hillerød. They then confer with the project psychiatrist in order to determine whether the potential subject meets the inclusion and exclusion criteria to a reasonable degree (see Exclusion 3). If necessary, the psychologists can recommend that Lone Fjorback, who is responsible for the welfare of the subjects, assesses an individual's psychiatric suitability for the project. Once 16 subjects have been approved, lots will be drawn for the two types of treatment. After this randomisation process (see also under the heading "Statistical considerations"), partners and subjects will be informed (see Figure S3).

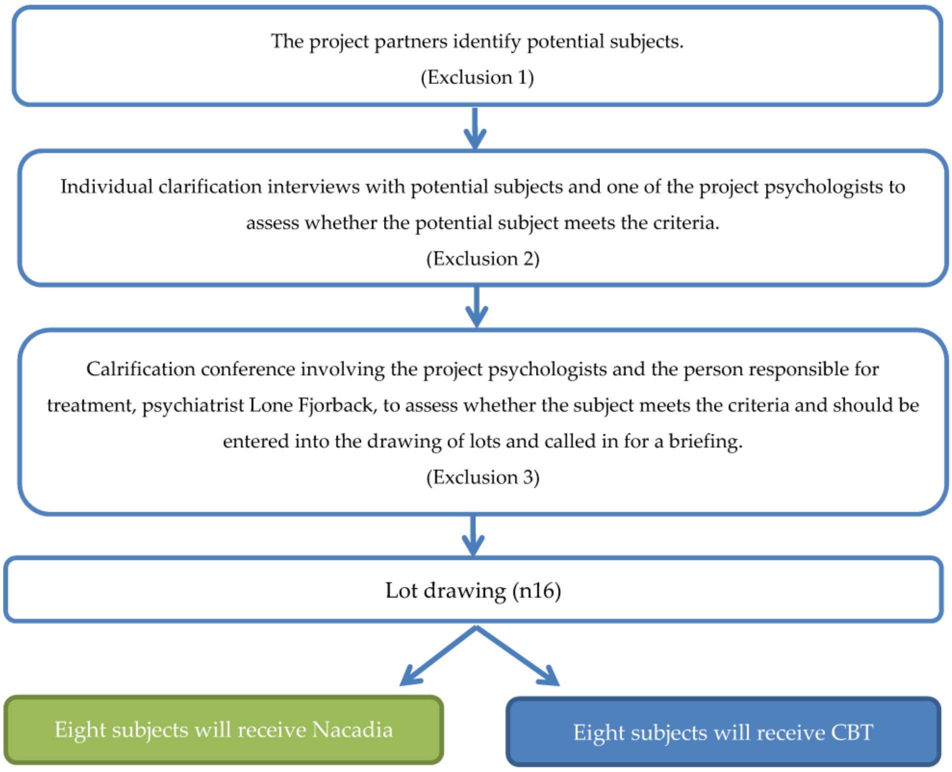

Figure S3. Potential routes into the project for the subjects.

5.3. Recruitment Procedure

The project is dependent on regular recruitment of test subjects, so that 16 subjects are available at each start-up date. A standard advertisement will be used for recruitment. The advertisement will be posted in the following newspapers: *Politiken*, *Frederiksberg Bladet*, and *Ugeavisen Hørsholm*. The advertisement text is true and fair and makes no distinction between the two types of treatment offered to the subjects. It refers to the project website, where further info is available (www.nacadia.dk).

6. Timetable for the Treatment

The treatment of group 1 starts on 5 August 2013. The potential subjects will be invited one by one to a clarification interview with the project psychologists. If additional exclusion is necessary, this will be done at the treatment conference. If there are spare places available, they will be offered to the other partners (Incita, Café Væksthuset and Topdanmark), after which the clarification of additional subjects will be conducted as soon as possible.

The CBT group schedule their own appointments with the psychologists. For the Nacadia therapy, the treatment days will be: 5, 7, 9, 12, 14, 16, 19, 21, 23, 26, 28, 30 August, 2, 4, 6, 9, 11, 13, 16, 18, 20, 23, 25, 27, 30 September, 2, 4, 7, 9, 11 October 2013.

In treatment week six, meetings will be held with coordinators. Each subject will be introduced to the idea of work placements, and preparation for the placements will start. For both types of therapy, follow-up will be after 1, 3, 6 and 12 months (for further information, see the research timetable). The coordinators monitor the subjects on their work placement, education or job.

The other group will follow the same rhythm as group 1 described above.

- Group 2 will start on 14 October 2013; clarification starts in September 2013,
- Group 3 will start on 3 February 2014; clarification starts in December 2013,
- Group 4 will start on 23 April 2014; clarification starts in March 2013,
- Group 5 will start on 12 August 2014, clarification starts in July 2014

7. Data Acquisition and Research Methodology

7.1. Primary Outcomes

The project has four primary outcomes:

1. Patient-related outcomes—quality of life (PGWB)
2. Function and health—relief of symptoms (Shirom-Melamed Burn-out Questionnaire)
3. Health-economics evaluation—Register data from Statistics Denmark

   A. Cost-efficiency:

      a. Reduced use of the healthcare system
      b. Resumption of work

   B. Patient-related outcomes (quality of life):

      a. Cost utility (expense effect/usability)
      b. Quality adjusted life years (EQ5D/PGWB)

4. Use of nature: Specifically for garden therapy, the patient's use of nature is a primary outcome.

The research involves both qualitative and quantitative methods, in the form of:

(a) landscape-architecture observation methods, landscape analyses, logbooks and semi-structured interviews;
(b) validated questionnaires and statistical data from central registers. Follow-up will take place immediately after the treatment and then at 3, 6 and 12 months.

7.2. Forms

The following validated forms are used in the research project and must be completed by all subjects:

1. Shirom-Melamed Burn-Out Questionnaire (SMBQ)—stress symptoms and burn-out
2. Psychological General Well-Being Questionnaire (PGWB)—general wellbeing
3. Euro Quality of Life (EQ5D)
4. Occupational Self Assessment (OSA) 1&2—activity self-assessment (things you do in everyday life in your home/place of work, etc.)
5. Sense of Coherence (SOC) 13-item scale—mastery and context
6. Client Satisfaction Survey (CSQ)—satisfaction with treatment
7. Functional Illness Checklist (FIC)
8. Background data: Information about age, gender, work, marital status, family relations, housing, leisure pursuits, previous medical and therapeutic treatment, current use of medicine or therapy, alcohol consumption, smoking habits, length of sick leave, duration of exposure to major stressors, attitude to the specific treatment and intervention, use of and preference for green areas.

The forms must be completed at the time of the clarification interview, at the end of the therapy, and 3, 6, and 12 months later. Assistance will be provided if the subjects need help reading or have difficulty understanding the questions, etc.

7.3. Databases

Data will be acquired using the subjects' civil registration numbers (before and after treatment). Background data will be acquired from Statistics Denmark. Additional data will be obtained from the following databases:

- The Medicine database
  - (medicine consumption)

- The DREAM database
    - (frequency of sick leave: how often and how long)
- The National Patient Registry and Health Insurance Registry
    - (use of the health system: how often and for how long).

7.4. Statistical Considerations

The effect of the treatments will be examined through statistical analyses of the change in the outcome measures (dependent variable). The two forms of treatment will be compared using multiple linear regression analysis, in which the change in the outcome measures will constitute the dependent variables, while the treatment groups will constitute the independent variables. Selected background data will act as a co-variable in relation to certain individual factors that are proven to benefit more from one type of treatment than the other.

The effect of the treatment will be measured by the change in the dependent variable (stress symptoms measured by SMBQ; well-being measured by PGWB; quality of life measured by EQ5D); and, the ratio of subjects who return to work.

The two types of treatment will be compared using a series of multiple linear regressions, in which the change in each of the dependent variables will be regressed on the two types of treatment as an independent variable. A calculation of robustness shows that a random sample of 40 subjects is enough to identify a mean difference of 9.2 between the two types of treatment (see Table S2). If gender is included as a control variable, a random sample of 40 subjects can identify a difference of 13.

Table S2. Power calculation.

**2-Sample *t*-Test**	
Test to see if the average of the two groups is the same (in relation to the differences)	
Alpha	0.05
Expected standard variance	14.5
Robustness of test	0.8
Significance limit	0.05
Sample size	40
Minimum identifiable difference	9.2

A person at the University of Copenhagen who has no contact with the test subjects will conduct the randomisation. The Excel function RAND (see Figure S4) is used to ensure a random spread of numbers between 0 and 1. The subjects' personal IDs will be entered in adjacent cells. Subjects with numbers below the median will be assigned to Nacadia therapy; those above, to CBT. The Excel spreadsheet can be adapted if there are fewer than 16 subjects.

Person ID	Tilfældige tal	Med eller ikke med
1	0,597519292	CBT
2	0,141317013	CBT
3	0,319607832	CBT
4	0,588555604	CBT
5	0,778022835	Nacadiaterapi
6	0,100542725	CBT
7	0,42369193	CBT
8	0,990046831	Nacadiaterapi
9	0,752425305	Nacadiaterapi
10	0,896417307	Nacadiaterapi
11	0,754381911	Nacadiaterapi
12	0,926248737	Nacadiaterapi
13	0,852189706	Nacadiaterapi
14	0,260578585	CBT
15	0,629702426	Nacadiaterapi
16	0,250231347	CBT

Figure S4. Example of randomisation in Excel.

7.5. Explorative Data Acquisition in Relation to Nacadia Therapy

The purpose of the explorative studies is to gain insight into the patients' preferences and use of the natural environment in the rehabilitation process, as well as to shed light on what it is about the therapy garden that produces the healing effect.

Observation of patients' use of Nacadia therapy (three hours/three times per group).

Semi-structured interviews carried out after a day of observation. The interview questions focus on the patients' preferences and use of the garden in relation to their healing process.

7.5.1. Observations

Prior to the observations, landscape-architecture analyses of the therapy garden are conducted, based on the perceived qualities of the environment. Analysis tool: "The Eight Perceived Sensory Dimensions (PSD)" [21,22].

Observations are made three times: 2, 6, and 9 weeks into the treatment. The purpose of the observations is to gain insight into whether the patients prefer specific areas and characteristics of the garden, and whether these preferences change during the therapeutic process or vary from person to person. Observation method: Records are kept of the behaviour of the individual patient and the group as a whole, stating the time, activity and location.

7.5.2. Logbooks

The subjects are invited to keep logbooks about their use, preferences and experiences of the garden.

7.5.3. Interviews

Each observation session will be followed by a semi-structured walk-and-talk interview with two patients per group. The interviews will last half an hour. The purpose of the interviews is to obtain insight into the patients' subjective experiences and how they use the garden during the rehabilitation process. The interviews function as a qualitative extension of the observations, and they help explore the roots of the patients' behaviour [23].

For the interviews, the garden therapists select two subjects from each group. The subjects are stratified based on the following considerations:

1. Good information flow, and
2. Extremes, from the most introverted to the most extroverted, and their use and perception of the garden.

7.5.4. Primary research question

Overall, the research can be divided into three parts; comparative impact studies, longitudinal impact studies, and an explorative study of the subjects' use of and preference for natural environments (see Figure S5).

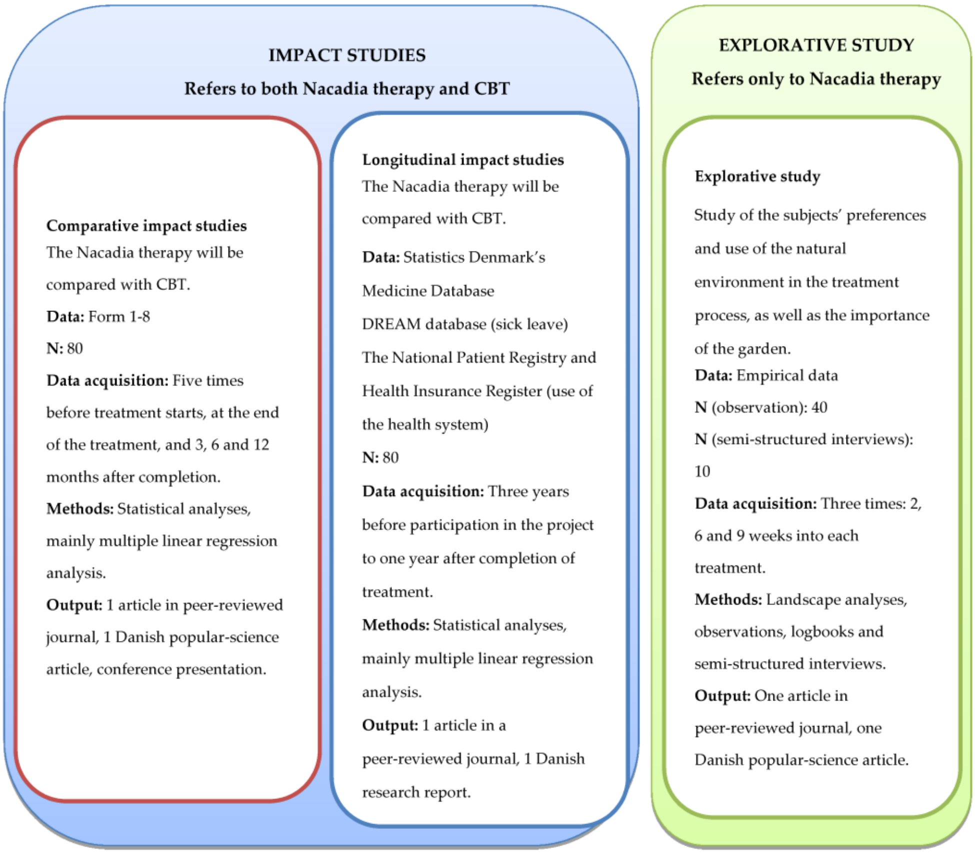

Figure S5. The three parts of the project research.

Main questions concerning Part 1—Comparative impact studies:

- Have the subjects' quality of life, function and health improved after treatment (studied before therapy (baseline), immediately afterwards, and then 3, 6 and 12 months after the end of the therapy)?
- Are there significant differences between the two forms of therapy in terms of quality of life, function and health?
- How satisfied are the subjects with the two types of treatment?

Main questions concerning Part 2—Longitudinal impact studies:

- Are the subjects in work/on sick leave 3, 6, and 12 months after completing the treatment? Is there a difference between the two types of treatment?
- What trends can be observed in the subjects' use of the healthcare system one year before participation and one year after the treatment has been completed? Is there a difference between the two types of treatment?
- What is the level of the subjects' medicine use before treatment, and at 3, 6 and 12 months after completion of treatment? Is there a difference between the two types of treatment?

Main questions concerning Part 3—Explorative study:

- Do the subjects use green environments differently after completing the treatment?
- Do the subjects express a preference for different places and activities in the garden at the end of the treatment than they did at the start?

- What qualities of the natural environment do the subjects express a preference for during therapy, and does this change over time?

7.6. Ethical Considerations

Ethical approval of the project has been sought from the Scientific Ethical Committee, Capital Region of Denmark (Ethical Approval Code: P.no. H-1-2013-038).

The Department of Geosciences and Natural Resource Management (IGN) is authorised to conduct register-based research. Notification of the acquisition of sensitive personal data, along with the subjects' consent, is submitted to Statistics Denmark via Høgni Kalsø Hansen, who is responsible for registry research at IGN.

The project has been registered with and approved by the Danish Data Protection Agency, which, in accordance with the Data Protection Act, permits the acquisition and processing of sensitive personal data for purely scientific or statistical purposes.

The information provided to the test subjects and the processing of personal data will be in accordance with the Data Protection Agency's terms and conditions for private research projects in relation to the duty of disclosure, subject insight and options for citing objections, as well as the retention and deletion of data.

Via their consent forms, the subjects agree to the acquisition and processing of sensitive personal data in accordance with Section 43 (1) of the Health Act. The subjects will be informed of the consequences of their participation in the project, including how they can access personal data stored on national registers, and their options for leaving the project. All of the subjects will be offered a copy of any data relevant to them, as well as the opportunity to discuss it with a psychotherapist involved in the study.

7.7. Expected Benefits for Test Subjects

After completing both types of treatment, it is expected that the subjects' quality of life will improve, their working capacity will increase, and their use of the healthcare system will decrease.

The subjects are expected to develop a psychological flexibility that will make them better able to handle personal challenges. This in turn will reduce the physiological follow-on effects of stress and re-establish a healthy balance in the nervous system. The subjects will also acquire "tools" that they can use to cope with stress situations in the future. This can be to the benefit of the subjects' families and workplaces.

In research terms, there is a demand for national and international evidence that garden therapy is as effective as recognised forms of therapy [24]. This project is unique due to its study of the importance of both the garden and the garden's design, which can be used to generate guidelines for evidence-based health design that can be implemented at both the national and international level.

7.7. Risks

As neither form of therapy involves the use of drugs, there is no immediate risk of adverse reactions for the project subjects. One potential disadvantage of both Nacadia therapy and CBT is that they can be demanding processes in which subjects are required to challenge themselves mentally/psychologically, and may falter or even regress. All of the subjects are informed of this in advance.

7.8. Process for Obtaining the Subjects' Consent

If somebody wants to participate in the project, they may, via their own doctor/psychiatrist or case worker (e.g., the insurance company's case officer), obtain information about the project, both verbally and in the form of a leaflet and letter, about what participation in the project will entail for the individual concerned. Each potential subject will also be given the pamphlet *Før du beslutter dig* (Before you decide), which provides information about their rights.

Those who wish to be involved provide their phone number and await a call from one of the project's referring psychologists. An appointment is made for the clarification interview in the psychologist's clinic where more information will be given. The subjects may, if they wish, be accompanied by a relative or other observer. All of the interviews take place in the psychologists' clinics, the therapy garden, or Nacadia's office—the aim of which is to ensure that the interviews are secure and undisturbed. After the clarification interview, the project's psychologists confer with the person with medical responsibility for the project in order to determine who should be invited to participate. Selected subjects will be invited to an information interview. The invitation will be sent along with a stamped addressed envelope and a consent form. Subjects will have at least 24 hours to think about whether they want to proceed.

7.9. Insurance

The University of Copenhagen is self-insured and has responsibility for the treatment of subjects in the project. The health treatment of the subjects is covered by patient insurance, cf. the Danish Act on complaints and compensation in the health service (consolidated act. No. 113 of 7 November 2011). The University of Copenhagen is also liable for damages under the general rules of Danish law on liability.

8. Timetable for the Research

8.1. Timetable for the Comparative Research Component

The research starts with the comparative research component. The research will follow the same procedure for all groups (see Table S3).

- The forms are completed a week before treatment starts.
- Week 10. During the last week of treatment, the forms are completed again (this time, the Client Satisfaction Questionnaire (CSQ) is also completed).
- Forms are again completed at 3, 6, and 12 months after the completed course of therapy. The Nacadia therapy subjects complete the forms in the garden. CBT subjects do so in the psychologists' waiting rooms.

Table S3. Timetable for the comparative research component.

Group	Form 1st Time	Form 2nd Time	Form 3rd Time (after 3 Months)	Form 4th Time (after 6 Months)	Form 5th Time (after 12 Months)
Group 1	Week 31 2013	Week 41 2013	Week 3 2014	Week 16 2014	Week 41 2014
Group 2	Week 41 2013	Week 51 2013	Week 12 2014	Week 25 2014	Week 51 2014
Group 3	Week 5 2014	Week 16 2014	Week 29 2014	Week 42 2014	Week 16 2015
Group 4	Week 16 2014	Week 26 2014	Week 40 2014	Week 2 2015	Week 26 2015
Group 5	Week 33 2014	Week 43 2014	Week 4 2015	Week 17 2015	Week 43 2015

Once all of the forms have been collected and scanned, a six-month phase of data analyses and article writing begins. This is expected to be completed on 31 April 2016.

8.2. Timetable for the Longitudinal Research Component

This research will start in Week 27, 2015, once all of the data has been collated. The data will then be analysed for six months on the basis of the study from the above-mentioned databases. The plan is that this work will be completed by 31 April 2016.

8.3. Timetable for the Explorative Research Component

Observations are made three times: 2, 6, and 9 weeks into the treatment. Each observational study is followed up by an individual semi-structured walk-and-talk interview with two patients per group (see Table S4).

Table S4. Timetable for the explorative research component.

Group	Data Acquisition 1	Data Acquisition 2	Data Acquisition 3
Group 1	Week 33 2013	Week 37 2013	Week 40 2013
Group 2	Week 43 2013	Week 47 2013	Week 50 2013
Group 3	Week 7 2014	Week 11 2014	Week 15 2014
Group 4	Week 18 2014	Week 22 2014	Week 25 2014
Group 5	Week 33 2014	Week 37 2014	Week 40 2014

8.4. Communication of the Outcomes

The project outcomes—positive, negative, and inconclusive—will be conveyed to an international audience via peer-reviewed articles in scientific journals and in presentations at international conferences. The results will be communicated to the Danish public in popular-science articles and in a report on the long-term effect.

8.5. Relevance and Target Group

There is a need for more knowledge about effective stress treatment in order to address the increase in stress-related health problems. In this light, the interdisciplinary meeting between research into nature, health, and landscape architecture has significant potential for stress treatment. The purpose of this project is to provide research-based knowledge about the relationship between nature and health through longitudinal studies of garden therapy for people affected by stress. At present, there is only limited evidence for this form of treatment from an international perspective, and no specifically Danish research.

The results from the project will add to existing knowledge about effective stress treatments and inspire therapists in the field. It is assumed, on the basis of previous research, that the results of the project will indicate that nature is a positive factor in stress treatment, and will therefore be of interest for local authorities' planning and management of green areas.

9. Project Organisation

The project has three overarching main players:

1. The Department of Geosciences and Natural Resource Management (IGN), University of Copenhagen
2. The Department of Psychology, University of Copenhagen
3. The Research Clinic for Functional Disorders, Aarhus University.

IGN is the project owner and has overall responsibility, which includes:

- ensuring that the project progresses as described in this description;
- ensuring that the therapists deliver the described treatment and that the therapy garden is maintained;
- ensuring that data is collated and analysed;
- ensuring that the project submits reports to the Tryg Foundation; and,
- ensuring that the project results are communicated to the international scientific community via scientific journals and presentations at international conferences, as well as to the Danish public in reports and popular-science articles.

Professor Ulrika K. Stigsdotter is project manager and is responsible for the research. Before the start of the project, an assistant professor in landscape architecture will be appointed to collate data for the project. Other participants will be involved in the project's research component as per the Vancouver system. Dorthe Djernis will be responsible for the implementation (co-ordination) of the Nacadia therapy, and will be head garden therapist for the project. Psychologist Eva Broby and Arboretum Supervisor Ole Byrgesen are also associated with the Nacadia therapy part of the project. Associate Professor Helmer Bøving Larsen, of the Department of Psychology, University of

Copenhagen, will be responsible for the implementation (co-ordination) of CBT. The psychologists Helle Engegaard and Katrine Bonnevie have been linked to this type of therapy (see Figure S6).

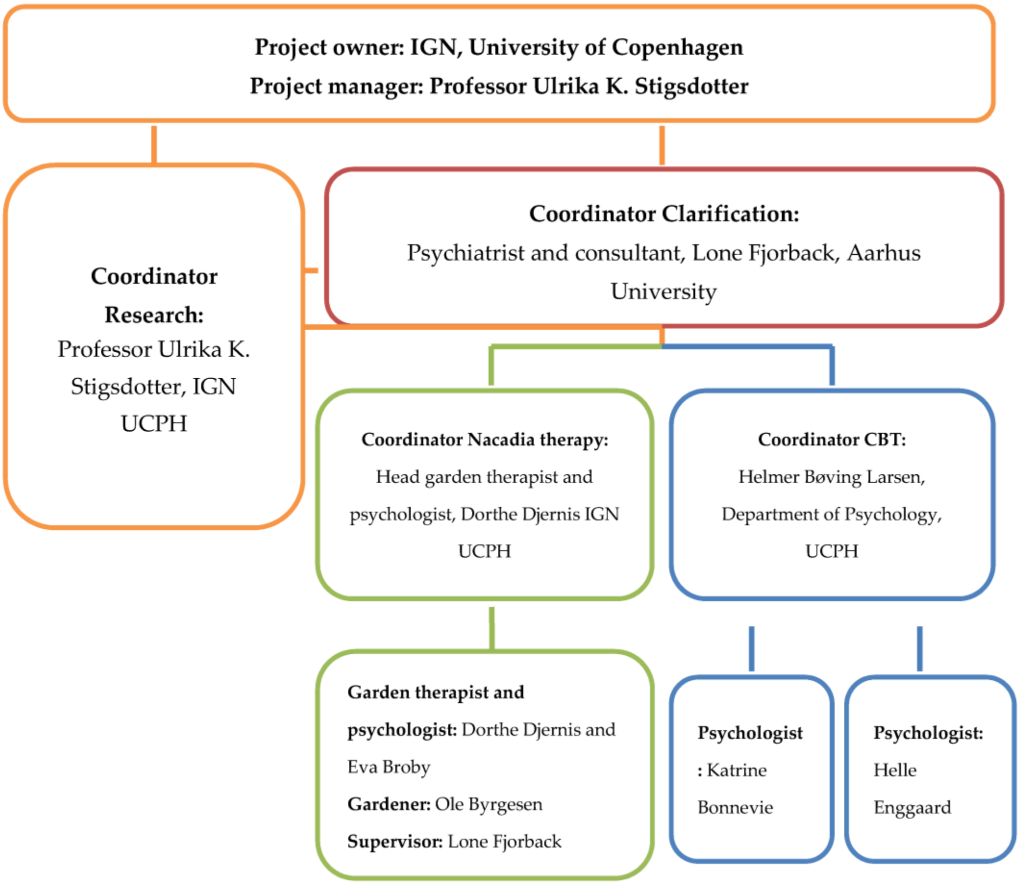

Figure S6. Schematic presentation of the project organisation.

9.1. Budget

In 2006, Professor Ulrika K. Stigsdotter initiated the Nacadia therapy garden project. The garden, which opened in 2011, cost DKK 6,350,000, and was funded by Realdania, the Obel Family Foundation and G.B. Hartmann's Family Foundation, as well as the University of Copenhagen. The NEST project has a budget of DKK 3,000,000 over a three-year period. In 2012, an updated version of the NEST application, with Professor Stigsdotter as project applicant/manager, received a grant of DKK 3,000,000 from the Tryg Foundation.

Table S5 presents the project budget.

Table S5. Project budget.

Stakeholders	Items:	DKK
	**Treatment**	**Total**
IGN, University of Copenhagen	Nacadia therapy (DKK 810,000.00)<br>Gardener (DKK 180,000)<br>Preliminary conversation, clarification, journal-keeping (DKK 90,000)	1,080,000
The Department of Psychology, University of Copenhagen<br>Research Clinic for Functional Disorders, Aarhus University	CBT (DKK 711,000)<br>Supervision (DKK 50,000)<br>Preliminary conversation, clarification, journal-keeping (DKK 90,000)<br>Training and clarification conferences (DKK 112,000)<br>Supervision (DKK 80,000)	1,043,000
	Preparation and administration	
IGN and the Department of Psychology, University of Copenhagen	Marketing (DKK 77,648)<br>Coordinator functions (DKK 35,961)	212,609

	Research	
IGN, University of Copenhagen	Comparative impact studies (DKK 316,115)<br>Longitudinal impact studies (DKK 190,000)<br>Explorative study (DKK 158,276)	664,391
	Unforeseen expenses (DKK 99,000)	
TOTAL		3,000,000

References

1. Annerstedt, M.; Wahrborg, P. Nature assisted therapy: Systematic review of controlled and observational studies. *Scand. J. Public Health* **2011**, *39*, 371–388.
2. Hartig, T.; van den Berg, A.E.; Hagerhall, C.M.; Tomalak, M.; Bauer, N.; Bell, R.H.; Ojala, A.; Syngollitou, E.; Carrus. G.; Herzele, A. et al. Health benefits of natural experience: Psychological, social and cultural processes. In *Forest, Trees and Human Health*; Nilsson, K., Sangster, M., Gallis., C., Hartig, T., de Vris, S., Seeland, K., Schipperijn, J., Eds.; Springer: New York, NY, USA, 2011; pp. 127–169.
3. Stigsdotter, U.K.; Grahn, P. What Makes a Garden a Healing Garden? *J. Ther. Hortic.* **2002**, *13*, 60–69.
4. Hartig, T.; Cooper Marcus, C. Healing gardens: Places for nature in healthcare. *Lancet* **2006**, *368*, 36–37.
5. Cooper Marcus, C.; Barnes, M. (Eds.) *Healing Gardens: Therapeutic Benefits and Design Recommendations*; Wiley & Sons: New York, NY, USA, 1999
6. Bellarosa, C.; Chen, P.Y. The effectiveness and practicality of occupational stress management interventions: A survey of subject matter expert opinions. *J. Occup. Health Psychol*. **1997**, *2*, 247–262.
7. Edwards, D.; Burnard, P. A systematic review of stress and stress management interventions for mental health nurses. *J. Adv. Nurs.* **2003**, *42*, 169–200.
8. Reynolds, S. Interventions: What works, what doesn´t? *Occup. Med.* **2000**, *50*, 315–319.
9. Van der Hek, H.; Plomp, H.N. Occupational stress management programmes: A practical overview of published effect studies. *Occup. Med.* **1997**, *47*, 133–141.
10. Hurrell, J.J., Jr.; Murphy, L.R. Occupational stress intervention. *Am. J. Ind. Med.* **1996**, *29*, 338–341.
11. Murphy, L.R. Stress management in work settings: A critical review of the health effects. *Am. J. Health Promot.* **1996**, *11*, 112–135.
12. Netterstrøm, B.; Conrad, N. *The Relationship between Work-Related Stressors and the Development of Mental Disorders Other than PTSD*; A reference document on behalf of the Danish Work Environment Research Fund; Danish Work Environment Research Fund: København, Switzerland, 2007
13. McCraty, R.; Atkinson, M.; Tomasino, D. Impact of a workplace stress reduction program on blood pressure and emotional health in hypertensive employees. *J. Altern. Complement. Med.* **2003**, *9*, 355–369.
14. Van der Klink, J.J.; Blonk, R.W.B.; Schene, A.H.; Dijk F.H.J.V. Reducing long-term sickness absence by an activating intervention in adjustment disorders: A cluster randomised controlled design. *Occup. Environ. Med.* **2003**, *60*, 429–437.
15. Niuwenhuijsen, K.; Verbeek, J.H.; de Boer, A.G.; Blonk, R.W.; van Dijk, F.J. Supervisory behaviour as a predictor of return to work in employees absent from work due to mental health problems. *Occup. Environ. Med.* **2004**, *61*, 817–823.
16. Perski, A. Rehabilitation of stress-related diseases goes through different phases and is often long-lasting [in Swedish]. *Lakartidningen* **2004**, *101*, 1292–1294.
17. Gonzalez, M.T.; Hartig, T.; Patil, G.G.; Martinsen, E.W.; Kirkevold, M. Therapeutic horticulture in clinical depression: A prospective study of active components. *J. Adv. Nurs.* **2010**, *66*, 2002–2013.
18. Tenngard Ivarsson, C. On the Use and Experience of a Health Garden. PhD Dissertation, Swedish University of Agricultural Sciences, Uppsala, Sweden, 2011.
19. Grahn, P.; Ottosson, Å. (Eds.). *Alnarpsmetoden. Trädggårdsterapi: Att la Hjälp av Naturen vid Stress och Utmattning*; Bonnier Existens: Lund, Sweden, 2010.
20. Fjorback, L.O. *Mindfulness manual* [in Danish]. PsykiatriFonden, Copenhagen, Denmark, 2009.
21. Stigsdotter, U.K.; Grahn, P. Stressed individual's preferences for activities and environmental characteristics in green spaces. *Urban For. Urban Green.* **2011**, *10*, 295–304.
22. Grahn, P.; Stigsdotter, U.K. The relation between perceived sensory dimensions of urban green space and stress restoration. *Lands. Urban Plan.* **2010**, *94*, 264–275.
23. McNamara, C. General Guidelines in Conducting Interviews. Authenticity Consulting LLC.: Minneapolis, MS, USA, 1999.

24. Stigsdotter, U.K.; Palsdottir, A.M.; Burls, A.; Chermaz, A.; Ferrini, F.; Grahn. P. Nature-Based Therapeutic Interventions. In *Forests, Trees and Human Health*; Nilsson, K., Sangster, M., Eds.; Springer: Heidelberg, Germany, 2011; Chapter 11, pp. 309–342.

© 2018 by the authors. Submitted for possible open access publication under the terms and conditions of the Creative Commons Attribution (CC BY) license (http://creativecommons.org/licenses/by/4.0/).
